# Novel Function of *Distal-less* as a Gap Gene during Spider Segmentation

**DOI:** 10.1371/journal.pgen.1002342

**Published:** 2011-10-20

**Authors:** Matthias Pechmann, Sara Khadjeh, Natascha Turetzek, Alistair P. McGregor, Wim G. M. Damen, Nikola-Michael Prpic

**Affiliations:** 1 Georg-August-Universität Göttingen, Johann-Friedrich-Blumenbach-Institut für Zoologie und Anthropologie, Abteilung für Entwicklungsbiologie, GZMB Ernst-Caspari-Haus, Göttingen, Germany; 2 Institut für Populationsgenetik, Veterinärmedizinische Universität Wien, Vienna, Austria; 3 School of Life Sciences, Oxford Brookes University, Oxford, United Kingdom; 4 Friedrich-Schiller-Universität Jena, Department of Genetics, Jena, Germany; Janelia Farm Research Campus, Howard Hughes Medical Institute, United States of America

## Abstract

Despite many aspects of the regulation of segmentation being conserved among arthropods, the evolution of novel gene functions has played an important role in the evolution of developmental regulation and the emergence of new segmental structures. Moreover the study of such novel gene functions can be informative with respect to the patterns and direction of evolutionary changes in developmental programs. The homeobox gene *Distal-less* (*Dll*) is known for its conserved function in appendage development in metazoans. In arthropods, *Dll* is required for the specification of distal appendage structures. Here we describe a novel and unexpected role of *Dll* in the spider *Achaearanea tepidariorum*. We detect *At*-*Dll* transcripts not only in the appendages, but unexpectedly also in an anterior domain during early development, prior to the specification of the limb primordia. A similar early *Dll* domain is present in the distantly related spider *Pholcus phalangioides*. In *A. tepidariorum* this early *At*-*Dll* expression is required for head segmentation. RNA interference results in spiders that lack either the first or the first and the second walking leg segments. The early *At-Dll* expression is also required for the activation of the segment polarity genes *engrailed* and *hedgehog* in this region. Our work identifies the *Distal-less* gene as a novel factor in anterior spider segmentation with a gap gene-like function. This novel role of *Dll* is interesting because *Dll* expression is reduced in this region in crustaceans and the homologous insect segment, the mandible segment, does not express *Dll* and does not require this gene for patterning. We therefore discuss the possible implications of our results for understanding the evolution and diversification of the mandible segment.

## Author Summary

The development and segmentation of the head of the fly *Drosophila* is one of the best-studied examples of how tissues become genetically specified during embryonic development. However, the mechanisms for head segmentation vary considerably among the arthropods. This is on the one hand surprising because the head consists of the same series of segments in all arthropods. On the other hand, changes in gene regulatory networks are the basis for the evolution of novel morphologies and biodiversity. In this paper, we describe our study of the *Distal-less* gene in the spider *Achaearanea tepidariorum*. *Distal-less* is well-known for its function in appendage development, but here we show that in the spider it also has a novel function during head segmentation that is not found in *Drosophila* or other arthropods. In *Achaearanea* the *Distal-less* gene is necessary for the formation of the first walking-leg segment, which is homologous to the mandible segment of the head of other arthropods. Intriguingly, the mandible segment in other arthropods exhibits reduced or missing *Distal-less* expression. Thus, our results suggest that this difference in the role of *Distal-less* in the first walking-leg/mandible segment of spiders and other arthropods may underlie the diversification of this segment.

## Introduction

The genetic regulation of head segmentation in *Drosophila melanogaster* is one of the best-studied examples of how tissues become specified and patterned during early embryonic development [1], [2]. Moreover, some features of the *Drosophila* segmentation gene cascade are evolutionarily conserved among other arthropods [3]. However, although the arthropod head itself is morphologically an ancient and evolutionary conserved structure, composed of a protocerebral (or ocular) region followed by a series of five homologous segments [4], [5], there is increasing evidence that the upstream mechanisms regulating head patterning vary among arthropods [3], [6].

The formation of the head in *Drosophila melanogaster* begins with a maternal and early zygotic gradient system involving the proteins encoded by the *bicoid* (*bcd*), *hunchback* (*hb*), and *orthodenticle* (*otd*) genes [2], [3], [7]–[9]. These gradients provide a coordinate system for a large number of subordinate genes that further subdivide the head epidermis. Thus, the initial gradient system controls the final location of the segmental borders and the correct number of head segments. Surprisingly, although all insect heads have the same segmental composition, neither the *bcd* gene, nor the role and regulation of *hb* and *otd* is conserved in other insect species [3], [6]. It appears, therefore, that the evolutionarily conserved architecture of insect heads is produced by an as yet comparatively unexplained diversity of developmental genetic mechanisms. This phenomenon also extends to other arthropods: recent work in the spider *Achaearanea tepidariorum* demonstrated novel head formation mechanisms involving dynamic gene expression [10]. Understanding head development in arthropods will thus shed light on the flexibility and evolvability of the gene network underlying the patterning of this ancient and evolutionarily conserved structure.

Head segmentation mechanisms not only differ between species, but between adjacent head segments within a species. Developmental studies in *Drosophila* have shown that the embryonic head consists of two parts that use different mechanisms for segmentation [1], [11]. The anterior head part (or procephalon) uses a set of head gap genes to establish the location of the segments [7]–[9]. The posterior head part (or gnathocephalon) uses the pair-rule genes for segmentation and thus closely resembles the establishment of the trunk segments [2], [12]. The segment that abuts this border between the two segmentation mechanisms is the mandible segment. It is the anterior-most gnathocephalic segment that (together with the posterior part of the intercalary segment) receives input from both the head gap genes and the pair-rule genes. This integration of two developmental mechanisms is mediated by the gene *collier* (*col*; also known as *knot* (*kn*)), which acts specifically at the interface of the pro- and gnathocephalic head segmentation mechanisms [13]. Recent studies in spiders have shown that the spider head is also divided into an anterior and a posterior part distinguished by different segmentation mechanisms [10], [14], [15], but these segmentation mechanisms differ substantially from those in *Drosophila* and other insects. First, the pair-rule gene ortholog *hairy* is involved in the segmentation of both parts of the spider head. Second, the anterior head region (procephalon) of the spider employs a dynamic wave of gene expression for segmentation [10], a mechanism that is not used for anterior patterning in *Drosophila*. Establishment of the posterior head region of the spider (gnathocephalon) depends on a gap gene mechanism, more similar to *Drosophila* [14]. Third, the *col* gene is not expressed at the interface of the pro- and gnathocephalon in spider embryos [16]. Despite these differences, the spider homolog of the insect mandible segment, the first walking leg segment (L1), also develops at the interface of the two different segmentation mechanisms in the spider head. Thus, although the pro- and gnathocephalic segmentation mechanisms have diverged during spider and insect evolution, the role of the mandible segment as the interface of the two regions of the embryo where these mechanisms act is evolutionarily ancient. How then is the L1 segment generated in spiders?

The homeobox gene *Distal-less* (*Dll*) is well known for its evolutionarily conserved role in distal appendage formation [17], [18]. However, we also find that the *Dll* gene of *A. tepidariorum* (*At-Dll*) is expressed unexpectedly early in the L1 region, before the formation of the germ band and the specification of the appendages, and concurrent with the dynamic gene expression in the procephalon. This early expression of *Dll* is also seen in the spider *Pholcus phalangioides*, which is only distantly related to *A. tepidariorum*. We demonstrate in *A. tepidariorum* that this early expression of *Dll* in the L1 region is necessary for the specification of the entire L1 segment. Our results show that loss of *At-Dll* function leads to the complete loss of the L1 segment in adult spiders, and the loss of this segment in the embryo is preceded by the loss of segmentation gene expression and increased cell death. Thus, *At-Dll* functions similarly to the head gap genes that pattern the procephalic head segments in *Drosophila* [7], [8], implicating *At-Dll* as a novel head gap gene in spider head segmentation. Our results suggest that in contrast to the procephalic segments of this spider, the L1 segment is specified in a manner mechanistically similar to procephalic segment specification in *Drosophila* (i.e. involving the action of statically expressed head gap genes). The gene expression data from the cellar spider *P. phalangioides* suggest that the head gap gene role is more widely conserved in spiders. It is interesting to note that in insects and other mandibulate arthropods the expression levels of *Dll* in the homologous segment are reduced [19], [20] and we discuss possible implications of the divergent roles of *Dll* in this segment for the evolution of the mandible segment in the arthropods.

## Results

### Unexpected early *Dll* expression in the spider head

**Figure 1:**
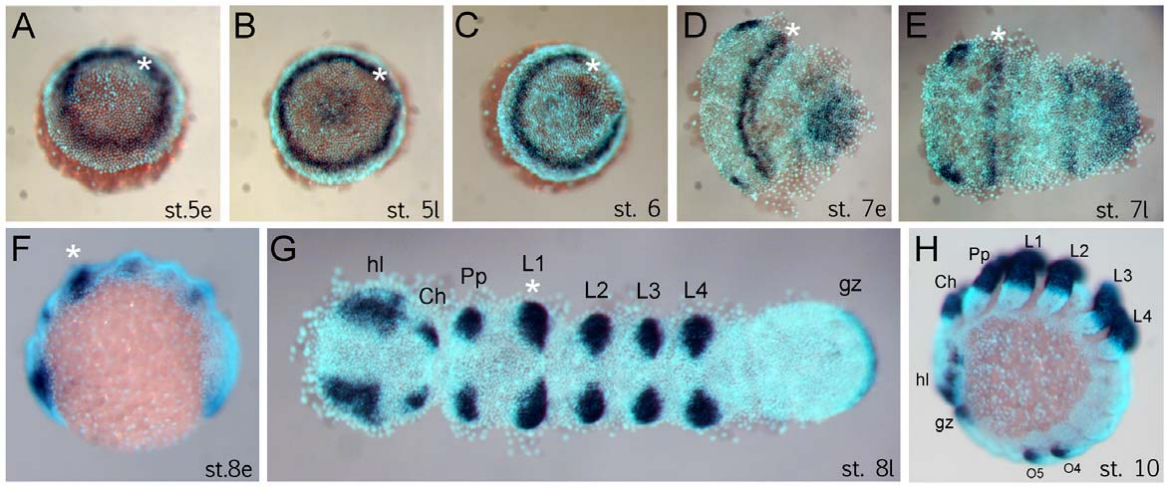
Expression of *Dll* in *Achaearanea tepidariorum* embryos. (A, B) Early ring of expression at early stage 5 (A) and late stage 5 (B). (C–E) Formation of the germ band. The *Dll* ring opens at stage 6 (C) and becomes an anterior stripe in the presumptive L1 region (D, E). (F–H) Late expression of *Dll* at early stage 8 (F), late stage 8 (G) and stage 10 (H) comprises additional domains in the appendages, head lobes, and growth zone. Abbreviations: Ch, chelicera; gz, growth zone; hl, head lobe; L, walking leg; O, opisthosomal segment; Pp, pedipalp. The asterisk marks the location of the early ring and its derivative head stripe in L1.

The anterior (“head”) segments of the spider *A. tepidariorum* are specified very early in embryonic development, at the germ disc stage when the embryo still has radial symmetry, and well before the transition into the bilateral symmetric germ band [10], [14], [21], [22]. The *Dll* gene of *A. tepidariorum* is strongly expressed in a static ring in presumptive anterior cells of the L1 region already at early stage 5 (staging after [21]) (Figure 1A). This is surprising because this expression occurs very early in development, long before the specification of the limb primordia, and concurrent with the general anterior-posterior patterning of the head region [10], [14]. The ring of *At-Dll* expression persists through late stage 5 (Figure 1B) and opens during the transition from germ disc to germ band formation at stage 6 (Figure 1C). Subsequently, as the germ band forms during early stage 7, the ring of expression is transformed into a broad stripe in the L1 segment (Figure 1D, 1E). In addition, a weaker stripe of expression is detected in the second walking leg segment (L2). Expression at later stages is found predominantly in the limb buds and other domains known for *Dll* homologs in other arthropods, (e.g. [17], [18]) (Figure 1F–1H).

### *Dll* is required for the formation of a head segment

**Figure 2:**
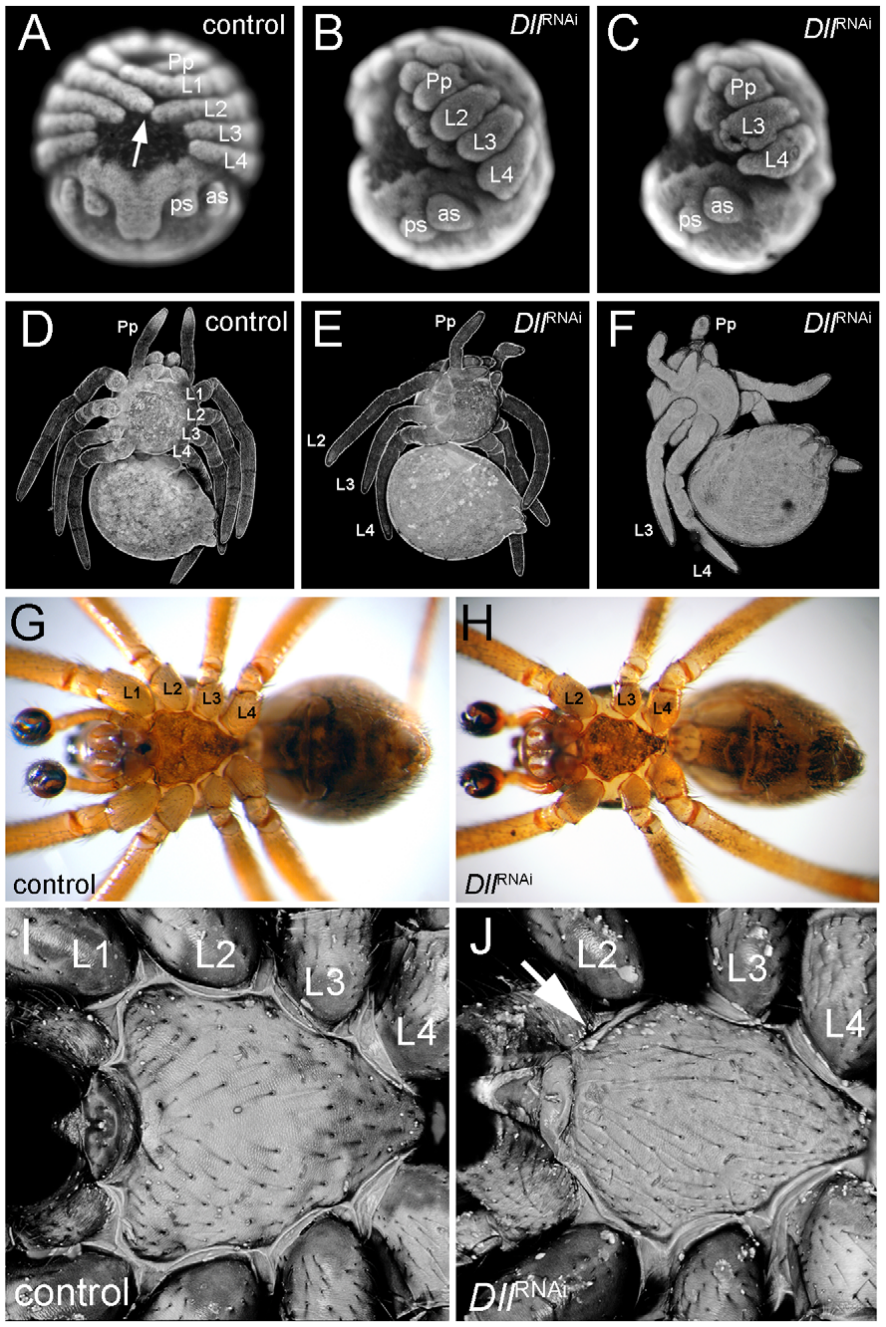
Phenotype of *At-Dll* RNAi animals. (A–C) Late inversion stage embryos of *Achaearanea tepidariorum*. In control embryos (A, embryo in a ventral view), the prosomal appendages are fully formed and the distal tips of L1 and L2 touch each other across the ventral midline (arrow). In strongly affected *Dll* RNAi embryos either L1 (B, embryo in lateral view) or L1 and L2 (C, embryo in lateral view) segments are missing and the remaining appendages are severely truncated. (D–F) Confocal scans of larvae. In control animals (D) all four walking leg segments are present. In *Dll* RNAi animals one (E) or two (F) leg segments are missing. (G, H) Ventral aspects of adult control male (G) and *Dll* RNAi male lacking the L1 segment (H). Note the fully sclerotized bulb of the pedipalps (dark colouring) indicating sexual maturity of the males. (I, J) Confocal scan of the sternum of adult control male (I) and *Dll* RNAi male (J). Note the missing anterior edge corresponding to L1 in the *Dll* RNAi animal (arrow in J). Abbreviations: L, walking leg; Pp, pedipalp; as, anterior spinneret; ps, posterior spinneret.

The unexpected early ring of *At-Dll* expression in the germ disc suggests that *At-Dll* plays a role in early head development in *A. tepidariorum*. We therefore tested the role of *At-Dll* in early head development using parental RNA interference (RNAi) (Table S1). RNAi led to a down-regulation of *At-Dll* transcripts in the cytoplasm in the early ring of anterior expression below the level of detection via in situ hybridisation (Figure S1A, S1B). In 64% of embryos, the RNAi effect was permanent, but in 19% the effect was no longer observed after stage 7 and thus led to a partial restoration of *At-Dll* transcripts in the subsequent stages of development (Figure S1D). In both cases, RNAi resulted in embryos lacking one entire leg-bearing segment, and in a few embryos with permanently down-regulated *At-Dll* expression, the loss of two entire leg-bearing segments was also observed. As expected, RNAi also affected appendage development resulting in severely truncated limbs in permanently affected embryos (Figure 2A–2C). In the other embryos, the development of the remaining legs was not affected owing to restoration of *At-Dll* expression in later stages. Importantly, this indicates that the patterning function of the early ring of *At-Dll* expression is required much earlier than the onset of appendage development and is necessary for segment formation.

While the permanently affected embryos did not survive, embryos where only the early *At-Dll* ring is down-regulated survived to reach the first larval instar. These juvenile spiders had only three pairs of walking legs (Figure 2E) or, in few cases, two pairs of walking legs (Figure 2F). The chelicerae, pedipalps, and the remaining legs were normal. The loss of some walking legs does not cause a discernible gap between the remaining appendages and this indicates that not only the legs, but also the corresponding segments are missing (see also below). The four-legged larvae did not survive, but the six-legged larvae survived and moulted normally and even reached adulthood (Figure 2G, 2H). Thus, under laboratory conditions the loss of a complete body segment does not appear to significantly compromise the viability and feeding behaviour of the animals (Video S1, Video S2).

### The L1 segment, the mandible segment homolog, is missing in *Dll* RNAi animals

In order to establish the identity of the missing walking leg segments, we compared morphological and structural landmarks between wild type and the six-legged adult *Dll* RNAi spiders. Adult spiders have a thick cuticle plate (the sternum) on the ventral side of their anterior body part (prosoma). The sternum in the wildtype has a characteristic shape with three pointed edges, the anterior most edge being the most pronounced (Figure 2I). In *A. tepidariorum*, like in other spider species [23], each walking leg segment except for L4 is represented on the surface of the sternum by a characteristic slit sense organ (Figure S2A). The slit sense organ of L1 and L3 is divided into two separate slits, whereas the slit sense organ of L2 comprises a group of three slits. In *At-Dll* RNAi animals the anterior most edge of the sternum is missing (Figure 2J, Figure S3A, S3B, S3C) and the bipartite slit sensory organ of L1 is lacking (Figure S2B). We conclude that the L1 segment is missing in these animals.

**Figure 3:**
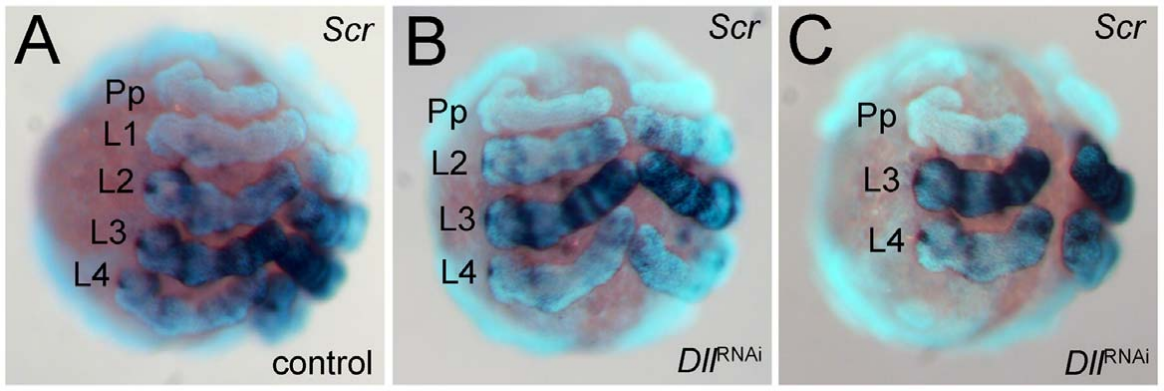
Expression of *Scr* in *At-Dll* RNAi embryos. (A–C) Ventral views of stage 10 embryos. The expression levels distinguish the four walking leg segments in control embryos (A) and identify the identity of the remaining leg segments in six-legged *Dll* RNAi embryos (B) and four-legged *Dll* RNAi embryos (C). Abbreviations: L, walking leg segment; Pp pedipalpal segment.

As an additional means of confirming the identity of the missing walking leg segments, we have used the expression of the Hox gene *Sex combs reduced* (*Scr*) as a marker [14]. This gene is expressed differentially in the four walking leg segments (L1 to L4) (Figure 3A). It is not expressed in L1, weakly expressed in L2 and L4, and strongly expressed in L3. The expression level of *Scr* thus allows the four walking leg segments to be distinguished in the embryo. In six-legged *At-Dll* RNAi embryos, the weakly expressing L2 and L4, and the strongly expressing L3 remain, thus confirming that L1 is missing (Figure 3B). In four-legged *At-Dll* RNAi embryos only the strongly expressing L3 and the weakly expressing L4 remain, thus confirming that L1 and L2 are missing (Figure 3C). There is no indication of homeotic transformation of the identity of the segments, rather the L1 or L1 and L2 segments, respectively, are absent. These results suggest that the early expression of *At-Dll* in the germ disc is required for the formation of the L1 and L2 segments and that the lack of early *At-Dll* expression leads to a gap gene-like phenotype in the anterior body region of *A. tepidariorum*.

**Figure 4:**
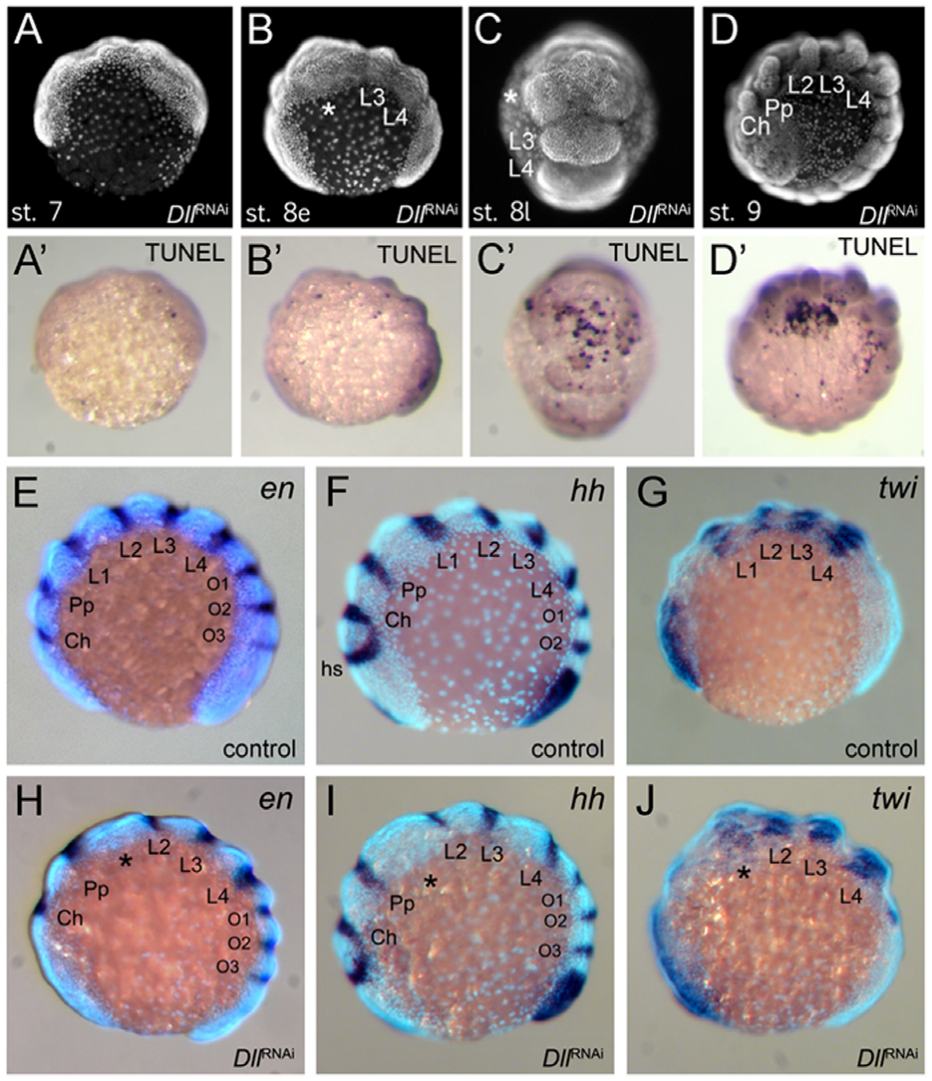
Effects of *At-Dll* RNAi. (A–D, A′–D′) Cell death during development of *Dll* RNAi embryos. (A–D) Morphology of the *Dll* RNAi embryos at stage 7 (A), early stage 8 (B), late stage 8 (C), and stage 9 (D) photographed under UV light. (A′–D′) These panels correspond to the panels A–D and show the same individuals as the panels A–D, except that the embryos were photographed under white light to document the TUNEL staining. (E–J) L1 specific elements of *en* and *hh* expression are deleted in *At-Dll* RNAi embryos. (E–G) Expression of *en* (E), *hh* (F), and *twi* (G) in control embryos. (H–J) Expression of *en* (H), *hh* (I), and *twi* (J) in *Dll* RNAi embryos. The asterisk denotes the malformed and bulged segment between pedipalpal and third walking leg segment. Abbreviations: Ch, cheliceral segment; L, walking leg segment; O, opisthosomal segment; Pp, pedipalpal segment.

To understand the mode of segment loss after early *At-Dll* loss, we have studied the development of the presumptive L1 and L2 region in RNAi embryos. During early germ band stages, when the segments first become morphologically visible, in *At-Dll* RNAi embryos, the body portion between the pedipalpal and L3 segment is bulged and larger than a single normal segment and likely consists of the cells normally comprising both the L1 and L2 segments (Figure 4A–4C). Cell death (Figure 4A′–4D′), however, does not occur in this bulged segment before late stage 8 when many dead cells were then detected in this segment (Figure 4C′). These data show that the loss of early *At-Dll* function leads to a malformation of the L1 and L2 segments and that a large portion of the cells in this malformed segment is later removed by cell death. Our results suggest that in the majority of cases the presumptive L2 cells remain and can still form this segment later during development. However, occasionally cell death apparently removes more cells in this region, resulting in the animals lacking both L1 and L2. Thus, the loss of L2 may not be a specific effect of the loss of the early *At-Dll* ring, but rather a secondary effect of occasional excessive cell death.

### *Dll* acts as a head gap gene in the spider

The *Dll* RNAi phenotype in *A. tepidariorum* is reminiscent of the mutant phenotypes of the head gap genes in *Drosophila* (e.g. *empty-spiracles* (*ems*) and *buttonhead* (*btd*)) [7], which also show the loss of anterior segments. One hallmark of these head gap genes is that they are required for the activation of the segment polarity genes (e.g. *engrailed* (*en*), *hedgehog* (*hh*)) in the particular head segments they specify [7], [8], [24]. We therefore investigated the impact of *At-Dll* RNAi on segment polarity gene expression in *A. tepidariorum*. We examined the expression of *hh* and *en*, both of which are normally expressed in single stripes in the posterior of each segment (Figure 4E, 4F). In *At-Dll* RNAi animals the expression of these genes was affected in the bulged segment between the pedipalpal and the L3 segment. The stripes of *hh* and *en* normally found in the posterior portion of L2 were still present, but the L1 stripe of both genes was missing (Figure 4H, 4I). These data indicate that the activation of these segmentation genes in the L1 segment directly or indirectly requires *At-Dll*. The lack of the L1 *en* and *hh* stripes in the bulged segment is not due to the absence of the cells that normally express these genes, because cell death in this segment occurs later, as described above (late stage 8; see Figure 4C). Moreover, the absence of *en* and *hh* expression does not reflect a general lack of gene expression in this malformed segment because the gene *twist* (*twi*), which is a mesoderm marker and not related to ectodermal segmentation or patterning [25] (Figure 4G), is still expressed in this bulged segment (Figure 4J).

Recently, it was found that the gene *hunchback* (*hb*) is crucial for the development of anterior segments in *A. tepidariorum* and is required for the formation of the L1, L2 and L4 segments [14]. Since the phenotypic effect of *At-hb* RNAi and *At-Dll* RNAi are similar with respect to the L1 segment and in part to the L2 segment, we reasoned that there might be an interaction between *At-hb* and *At-Dll* in the formation of these segments. However, we could not detect any obvious differences in *At-hb* expression in *At-Dll* RNAi embryos at the germ disc stage (Figure S4A, S4B), and the early ring of *At-Dll* expression was also expressed normally in *At-hb* RNAi embryos at the germ disc stage (Figure S4C, S4D). We conclude from these data that there is no clear or obvious interaction between *At-hb* and *At-Dll* during L1 and L2 segment development.

### Evidence for a head gap gene role of *Dll* in *Pholcus phalangioides*, a haplogyne spider

Our results show that *Dll* has a role in the formation and development of the L1 segment in *A. tepidariorum*, but is this a general mechanism for L1 formation in spiders or a novelty of this particular group of spiders like the role of *bicoid* in higher flies [26]? To answer this question, we studied *Dll* expression in the cellar spider *Pholcus phalangioides* (Figure S5A–S5E). The common house spider *A. tepidariorum* is a member of the Entelegynae that contain the vast majority of extant spiders. *P. phalangioides*, however, belongs to the Haplogynae, which is the sister group of the Entelegynae (Figure S6). Comparisons between *A. tepidariorum* and *P. phalangioides* can therefore reveal conserved features that trace from the common ancestor of entelegyne and haplogyne spiders.

The development of *P. phalangioides* differs somewhat from that of *A. tepidariorum*. The early stages of *P. phalangioides* also form a nearly round germ disc that undergoes a transition from a radial symmetric embryo to an axial symmetric embryo, which results in the formation of the germ band. In *A. tepidariorum* as well as in *P. phalangioides* the cumulus migrates from the centre of the germ disc to the rim of the disc, the future dorsal pole of the embryo [19], [27]. In *A. tepidariorum* the cumulus disappears after its migration, but in *P. phalangioides* the cumulus persists much longer and is still visible during early stages of germ band elongation (asterisk in Figure S5C). The earliest stages of *P. phalangioides* embryos that are accessible for in situ hybridisation are comparable to stage 6 or stage 7 embryos of *A. tepidariorum*. *Pp-Dll* in these embryos is expressed in an almost closed ring or stripe close to the anterior rim of the germ disc and forming germ band (Figure S5B, S5C). This expression is very similar to the early *Dll* ring that is observed in *A. tepidariorum* embryos (Figure S5B), and thus argues for the conservation of early *Dll* function in entelegyne and haplogyne spiders. The late pattern of *Pp-Dll* is also virtually identical to the pattern in *A. tepidariorum* (and other spiders) and includes strong expression in the cheliceres, pedipalps, and walking legs (Figure S5D, S5E).

## Discussion

While the last common ancestor of all arthropods is generally accepted to have been segmented and, moreover, had a segmented head, the molecular and genetic mechanisms for trunk segmentation and head segmentation are diverse in extant arthropods [6]. Understanding the genetic basis of head development in arthropods thus provides us with insights into the diversification of the underlying gene regulatory networks that nonetheless specify an evolutionarily conserved structure. Here we describe a novel function of the highly conserved *Dll* gene in head segmentation in the spider *Achaearanea tepidariorum* and we present evidence that this function is conserved in a second distantly related spider species, the cellar spider *Pholcus phalangioides*.

### A novel role for *Dll* as a head gap gene in *Achaearanea*

Previous results from the spider *A. tepidariorum* showed how positional values for procephalic segmentation genes are specified via the dynamic expression of the conserved head gene *otd* [10]. However, segmentation gene expression in the following walking leg segments (that constitute the gnathocephalon homolog of spiders) is static and does not depend on *otd* expression dynamics [10], [14]. The L1 segment has a special position in the spider head as it lies at the boundary between the procephalon and gnathocephalon that divides dynamic and static segmentation gene expression patterns. Our results identify a novel function of the appendage patterning gene *Dll* as a head gap gene in this particular head segment. An early static activation of *At-Dll* in L1 is necessary for the specification and formation of the L1 segment, and is required for the subsequent activation of the segmentation genes in this region. In these respects this early function of *At-Dll* is similar to the head gap genes that mediate procephalic segmentation in *Drosophila*, e.g. *ems* and *btd* [7], [8]. Remarkably, this head gap gene function of *At-Dll* represents a novel function of *Dll* and has not been observed in other arthropod groups. In addition, *Dll* function in *A. tepidariorum* is independent of the conserved early head factor *hb* and might act in parallel with this gene, whereas in *Drosophila* the head gap genes interact with *hb* [2], . Together, this indicates that the formation of the L1 segment in *A. tepidariorum* is mechanistically similar to procephalic segmentation in *Drosophila* (i.e. involving the action of statically expressed head gap genes), but employs some factors that are not used in segmentation in this insect. Indeed, it is currently unclear to what extent the mode of head development in *Drosophila* is representative of other arthropods. The specific functions of the maternal coordinate genes (e.g. *bcd*) are not conserved even within the higher flies (cyclorrhaphan dipterans) [26], [30], and in non-dipteran species the role of *bcd* appears to be assumed by different factors in different species [3], [6], [31]. The role of the head gap genes is only partially conserved in other insects, because recent results in the beetle *Tribolium castaneum* show that the *btd* gene does not have a role in head development [32], and the situation in non-insect arthropods is largely unknown. Thus, paradoxically, the evolutionarily conserved morphological structure of the arthropod head is produced by highly diverse molecular mechanisms. It is generally appreciated that the germ band stage in arthropod development represents the “phylotypic stage” for the phylum and functions as a bottleneck for the diverse developmental mechanism before and after that stage [33], [34]. Indeed, recent work has shown that the transcriptome that is active during the phylotypic stage is phylogenetically ancient and well-conserved [35], [36]. The morphology of the arthropod head is very similar at the germ band stage and thus is an integral part of the arthropod phylotypic stage. It thus appears that so long as the conserved transcriptome of the head is activated at the phylotypic stage, then what mechanisms actually activate this transcriptome does not really matter- hence the divergence in early head patterning mechanisms.

### Implications for understanding the evolution of the mandibular segment

Our results also have additional evolutionary implications. As mentioned above, the spider L1 segment is homologous to the insect mandibular segment [4], [5]. This segment in insects is remarkable for its complete lack of *Dll* expression, but yet produces the insect typical gnathal appendage, the mandible [20]. In other mandibulate arthropods (crustaceans, myriapods) reduced *Dll* levels are expressed during mandibular development [17], [18], [20], [37]. In contrast, our results show that *Dll* is broadly expressed in the mandibular segment homolog of spiders and has an important role in the formation of this segment.

The function of *Dll* has been studied previously in another spider, the Central American wandering spider *Cupiennius salei*, but no role of *Dll* in L1 segment formation has been observed [38]. The lack of gap-like phenotypes in *C. salei*, however, may have resulted from methodological limitations in this species because parental RNAi is not applicable in *C. salei* [38], and thus an early function of *Dll* might have been missed in the embryonic RNAi experiments. Indeed, we have performed embryonic RNAi for *Dll* in *A. tepidariorum* embryos identical to the RNAi procedure in *C. salei*, and obtained similar phenotypes as in *C.salei*: appendage phenotypes, but no gap phenotypes (Figure S7A–S7C). This class of phenotype is absent in parental RNAi experiments in *A. tepidariorum* where the appendage phenotypes (if present) are always associated with gap phenotypes (Table S1). These data suggest that the RNAi mechanism requires some time to develop the full effect after the application of the dsRNA, and thus only the parental injections provide the effect early enough, whereas the embryonic injections fail to interfere with the early expression of *Dll* and thus only lead to appendage phenotypes.

Early embryonic stages of *C. salei* are not accessible for in situ hybridisation, therefore it is not possible to analyse the early expression of *Dll* in *C. salei*. However, we could demonstrate that in another spider, the cellar spider *P. phalangioides*, *Dll* is expressed in a similar early anterior ring like in *A. tepidariorum*. *P. phalangioides* belongs to the Haplogynae, the sister group of the Entelegynae, the group to which *Achaearanea* belongs and that contains the majority of all extant spider species. These data suggest that the early gap-like function of *Dll* has already been present in the common ancestor of the entelegyne and haplogyne spiders. The only non-spider chelicerate that has been studied for *Dll* function is the mite *Tetranychus urticae*, but no gap-like phenotypes were reported [39]. The results in *T. urticae*, however, might be explained by the fact that mites show a derived chelicerate body plan with a mite-specific tagmosis of their anterior segments (these are fused into a structure called capitulum) and might therefore also be derived in terms of their anterior developmental mechanisms.

In summary, we present evidence for a gap-like function of *Dll* in entelegyne and haplogyne spiders in addition to its widely conserved role as an appendage gene. We propose the hypothesis that this dual role of *Dll* in spiders might have prevented the reduction of *Dll* expression levels in the spider homolog of the mandibular segment, because this would have led to severe and possibly deleterious phenotypes. By contrast, we propose that in the mandibulates, where *Dll* has no dual role, the reduction of *Dll* levels in the mandible segment only led to a distal reduction of the mandibular appendages as can be seen in Crustacea [17], [18], [37]. This novel type of distally reduced appendage has then further diversified also leading to the fully gnathobasic (i.e. lacking all distal elements) insect mandible. In this way, diversification of *Dll* function may have driven the diversification of mandible segment morphology and facilitated the evolution of the mandible as a tool to tap into new food resources.

Whether the gap-like role of *Dll* in the L1/mandible segment is the ancestral state for the arthropods or has evolved later in the chelicerate lineage is difficult to be determined in the absence of functional data from outgroups (e.g. onychophorans (velvet worms), tardigrades (water bears)). However, the presence of early *Dll* expression in the anterior body region of a mollusc [40], a hemichordate [41] and a cnidarian [42] suggests that *Dll* is ancestrally involved in anterior development, and it has been proposed recently that the majority of the leg patterning genes (including *Dll*) originally were involved in setting up the anterior-posterior head axis in the bilaterian animals and were only later co-opted for proximal-distal axis formation of the appendages [43].

## Materials and Methods

### Animal culture and gene cloning

Embryos and adults of *A. tepidariorum* were obtained from our laboratory stocks in Göttingen. A partial sequence of the *A. tepidariorum Dll* gene was cloned previously [44]. Additional sequence was obtained by analysing the embryonic transcriptome (sequenced from RNA isolated from stages 1 to 10) and by RACE PCR using the SMART RACE cDNA Amplification Kit (Clontech Laboratories, Inc.) and the following primer sets:

At-Dll-5′RACE-1: GGCCGAGAGTTGTGGGCTGAGGCG;

At-Dll-5′RACE-2: CTGGGCATGGTGGGGCATACCCGC;

At-Dll-3′RACE-1: CTCATCCGTACCTGGGTTCGTATCCGG;

At-Dll-3′RACE-2: CCGGCTGTCCGCCATGTCCATCACC.

The full *At-Dll* gene sequence has been deposited with the EMBL sequence database under accession number FM876233.

Adult females of *P. phalangioides* were collected from several cellars in Göttingen, Frankfurt am Main and Munich. Embryos were fixed as described previously [45].

A fragment of the homeobox of the *P. phalangioides Dll* gene was isolated using the primers eDP-fw, eDP-bw, iDP-fw, and iDP-bw as described previously [46]. RACE-PCR to obtain the full *Pp-Dll* sequence was performed using the primers Pp-Dll-5′RACE (CCA GTG ATG CGG CGA GTT CGG CTC) and Pp-Dll-3′RACE (GGT TTC AAA GGA CGC AGT ACC TGG CGC). The full *Pp-Dll* sequence has been deposited with the EMBL sequence database under accession number HE585694.

### RNA interference

Parental RNAi was performed as described previously [47] with minor modifications. To exclude off-target effects caused by the dsRNA injections, either the full length *At-Dll* fragment or a 1260 bp fragment, both including the homeobox, or two not overlapping fragments of 305 bp and 360 bp, both excluding the homeobox, were injected independently of each other into adult spider females. All injections led to identical phenotypes. For the knock-down of *hunchback*, dsRNA of a 1015 bp fragment was injected and led to the same phenotypes as described previously [14]. Control spiders were injected with water.

Embryonic RNAi was performed in *A. tepidariorum* by injections into the perivitelline space as described for *Cupiennius salei* [38]. *A. tepidariorum* embryos were injected at stage 4, being the earliest stage that has a perivitelline space that is wide enough for injections, thus sticking to the *Cupiennius* protocol [38] as closely as possible.

### In situ hybridisation, DNA labeling, imaging

In situ hybridisation, TUNEL and nuclear staining were performed as described previously [44], [48], [49] with minor modifications. For the TUNEL experiments both negative and positive controls were performed as previously described [49] to confirm the specificity of the obtained stainings (Figure S8A–S8D). Images of spider embryos or adult spiders were either captured with a Zeiss Axioplan-2 microscope or with a Leica dissection microscope equipped with an Intas digital camera and UV-light. Confocal z-stacks of spider larvae and of the sternum of adult spiders were captured using a Zeiss LSM 510. Before scanning the animals were heat fixed, embedded in Voltalef H10S oil and covered with a cover slip.

## Supporting Information

Figure S1 Confirmation of the RNAi with the expression of *At-Dll*. (A, A′) Detection of *At-Dll* transcripts in the early stripe in a stage 6 control embryo (A) and detailed magnification in (A′). Transcripts are detected in the nuclei and the cytoplasm. (B, B′) Detection of *At-Dll* transcripts in a stage 6 *At-Dll* RNAi embryo (B) and detailed magnification in (B′). Nascent *At-Dll* transcripts are detected in the nucleus, but no transcripts are present in the cytoplasm. (C, D) Expression of *At-Dll* in control (A) and *At-Dll* RNAi animals (B) at late stage 8. While expression of *At-Dll* remains permanently downregulated by RNAi in the majority of embryos, in some embryos expression reappears (D), leading to normal expression in later stages. Abbreviations: Ch, cheliceral segment; L, walking leg segment; Pp, pedipalpal segment.
(TIF)

Figure S2 Slit sense organs on the sternum of adult male animals. (A) Control animal. (B) *Dll* RNAi animal. The slit sense organs are emphasized in green colour. The sense organ of the first walking leg segment (denoted by the white circle) is lacking in *At-Dll* RNAi animals.
(TIF)

Figure S3 Shape comparison of the sternum in wildtype and *Dll* RNAi animals. (A, B) Adult wildtype and *Dll* RNAi spiders (same images as in Figure 2G, 2H) where the sternum has been marked in colour (red and blue, respectively). (C) Alignment of the shape of the *Dll* RNAi sternum (blue) with the wildtype sternum (red) reveals that the posterior edges (edges 2 and 3) align well, but edge 1 is only present in the wildtype sternum. Abbreviations: L, walking leg.
(TIF)

Figure S4 No interaction between *At-Dll* and *At-hb*. (A, B) Early (stage 6) expression of *At-hb* is virtually identical in control (A) and *At-Dll* RNAi animals (B). (C, D) Early (stage 6) expression of *At-Dll* is virtually identical in control (C) and *At-hb* RNAi animals (D). The embryos have also been stained with a probe against *orthodenticle* (*At-otd*) to mark the anterior rim of the embryo. Abbreviations: Ch, cheliceral segment; L, walking leg segment; Pp, pedipalpal segment.
(TIF)

Figure S5 *Dll* expression in *Pholcus phalangioides* embryos. (A) An adult *Pholcus phalangioides* female. (B) Several embryos of *P. phalangioides* stained for *Pp-Dll* transcripts. The embryos are in a developmental stage that is comparable to stage 6/7 of *Achaearanea tepidariorum* embryos. For comparison, an embryo of *A. tepidariorum* at stage 6 (denoted by the asterisk) and stained for *At-Dll* transcripts has been placed next to the *P. phalangioides* embryos. Similar to the expression of *Dll* in *A. tepidariorum* also *Pp-Dll* is expressed in an early anterior ring/stripe like domain. (C) Close-up of a *Pp-Dll* stained *P. phalangioides* embryo. The asterisk marks the cumulus and the white dotted line indicates the border between the embryonic and the extra-embryonic cells. Note the *Pp-Dll* expression stripe that is close to the anterior end of the embryo. (D, E) Late embryonic *Pp-Dll* expression pattern at the beginning of germ band inversion (D) and at the end of inversion (E). *Pp-Dll* transcripts are detected in the distal parts of all developing appendages. This late expression is virtually identical to *Dll* expression of other spider species [38], [44], [50]. Abbreviations: ch, cheliceral segment; L, walking leg segment; pp, pedipalpal segment; O, opisthosomal segment; A, anterior end; P, posterior end.
(TIF)

Figure S6 Overview of spider phylogeny. Spiders (Araneae) comprise a few primitive clades (Mesothelae, Mygalomorphae) and the speciose clade Neocribellatae that comprises the vast majority of all extant spiders. This clade consists of two sister groups, Haplogynae and Entelegynae. So far, all spider species (except for *Acanthoscurria geniculata* [44]) used for gene expression or gene function studies belong to the Entelegynae. The cellar spider *Pholcus phalangioides* is the only studied member of the Haplogynae. The similarities in early *Dll* expression in *A. tepidariorum* and *P. phalangioides* indicate that this patterning function of *Dll* was already present before the split of Haplogynae and Entelegynae. The tree is simplified after [51]. Only representative well-known families (where English common names were available) are shown, and examples (genus names) are given when representatives of the group have been used in gene expression/function studies. The red dots denote the placement of *Achaearanea* and *Pholcus* in the phylogenetic tree.
(TIF)

Figure S7 Embryonic RNA interference with *At-Dll*. (A) Control embryo injected with water. (B) After embryonic RNAi (eRNAi) with *At-Dll* all appendages are present but reduced in size. While parental RNA interference with *At-Dll* affects the early (segmental) and late (appendage) gene function (compare to Figure 2), embryonic RNAi only affects the development of the appendages. This result is consistent with the findings in *Cupiennius salei* in which eRNAi with *Cs-Dll* is leading to appendage defects, but no segment loss has been observed [38]. (C) Summary of the eRNAi results in *A. tepidariorum*. The phenotype was assessed at the late inversion stage. “Complete phenotype” refers to embryos with all appendages reduced in size. “Mosaic phenotype” refers to embryos with only some appendage reduced in size. “Developmental arrest” refers to embryos that showed some development, but died before reaching the late inversion stage, whereas “dead” refers to embryos that died at stage 4 and thus at or shortly after injection. “Unspecific” refers to embryos that reached the late inversion stage, but showed injection artefacts already known from eRNAi experiments in *Cupiennius salei* (i.e. malformation of body parts caused by unintentional injuries during injection). Abbreviations: Ch, cheliceral segment; L, walking leg segment; Pp, pedipalpal segment.
(TIF)

Figure S8 Confirmation of specific TUNEL staining after parental *At-Dll* knockdown. (A) An *At-Dll* pRNAi embryo showing enhanced cell death within the affected L1/L2 region. Please note that the L1 segment is already deleted in this embryo. (B) Only a few apoptotic cells are marked in the wild type control TUNEL staining. (C) In the negative control water instead of terminal deoxynucleotidyl transferase was added to the labelling reaction. (D) In the positive control the embryos were treated with DNase I before dig-UTP labelling [49]. Abbreviations: Ch, cheliceral segment; L, walking leg segment; Pp, pedipalpal segment.
(TIF)

Table S1 Summary of the *At-Dll* RNAi phenotypes. Adult females were injected five times (over a period of 10 days) with 1.5 µl dsRNA (3.8 µg/µl). Control females were treated the same as experimental animals, but injected with water. Female *A. tepidariorum* produce several cocoons during their lifetime. For this summary, embryos of cocoons 2 and 3 have been pooled and randomly chosen embryos have been assessed in detail for morphology and *At-Dll* expression.
(DOC)

Video S1 Prey-catching behaviour of a wildtype *A. tepidariorum*. The spider has caught a *Drosophila* fly and immobilizes it using silk threads. The silk threads are mainly manipulated with the legs of the L4 segment. The legs of the different walking leg segments L1 to L4 are highlighted in red in the final frames of the movie.
(MOV)

Video S2 Prey-catching behaviour of an *At-Dll* RNAi *A. tepidariorum*. The spider has caught a *Drosophila* fly and immobilizes it using silk threads. Like the control spiders, the RNAi spiders also manipulate the silk threads mainly with the legs of the L4 segment. There is no significant difference in this behaviour between control and RNAi spiders, despite the latter are lacking the L1 leg pair. The legs of the different walking leg segments L2 to L4 are highlighted in red in the final frames of the movie.
(MOV)
