# Supplementary figures and images for: Novel Function of Distal-less as a Gap Gene during Spider Segmentation
Source: PLoS Genet. 2011 Oct 20;7(10):e1002342. doi: 10.1371/journal.pgen.1002342 (PMC3197691; doi:10.1371/journal.pgen.1002342)

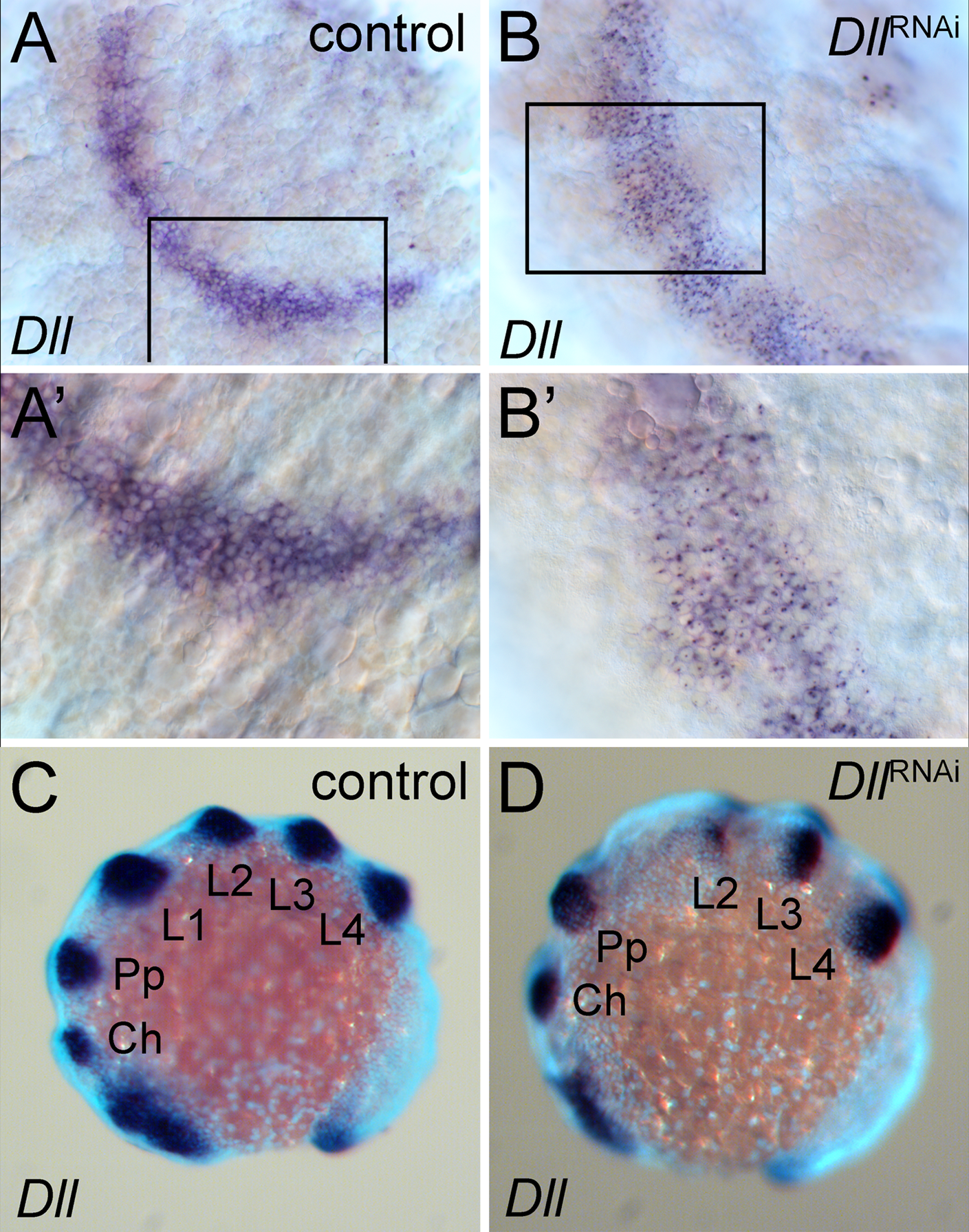

Supplement: Figure S1 — Confirmation of the RNAi with the expression of At-Dll. (A, A′) Detection of At-Dll transcripts in the early stripe in a stage 6 control embryo (A) and detailed magnification in (A′). Transcripts are detected in the nuclei and the cytoplasm. (B, B′) Detection of At-Dll transcripts in a stage 6 At-Dll RNAi embryo (B) and detailed magnification in (B′). Nascent At-Dll transcripts are detected in the nucleus, but no transcripts are present in the cytoplasm. (C, D) Expression of At-Dll in control (A) and At-Dll RNAi animals (B) at late stage 8. While expression of At-Dll remains permanently downregulated by RNAi in the majority of embryos, in some embryos expression reappears (D), leading to normal expression in later stages. Abbreviations: Ch, cheliceral segment; L, walking leg segment; Pp, pedipalpal segment. (TIF) [file pgen.1002342.s001.tif]

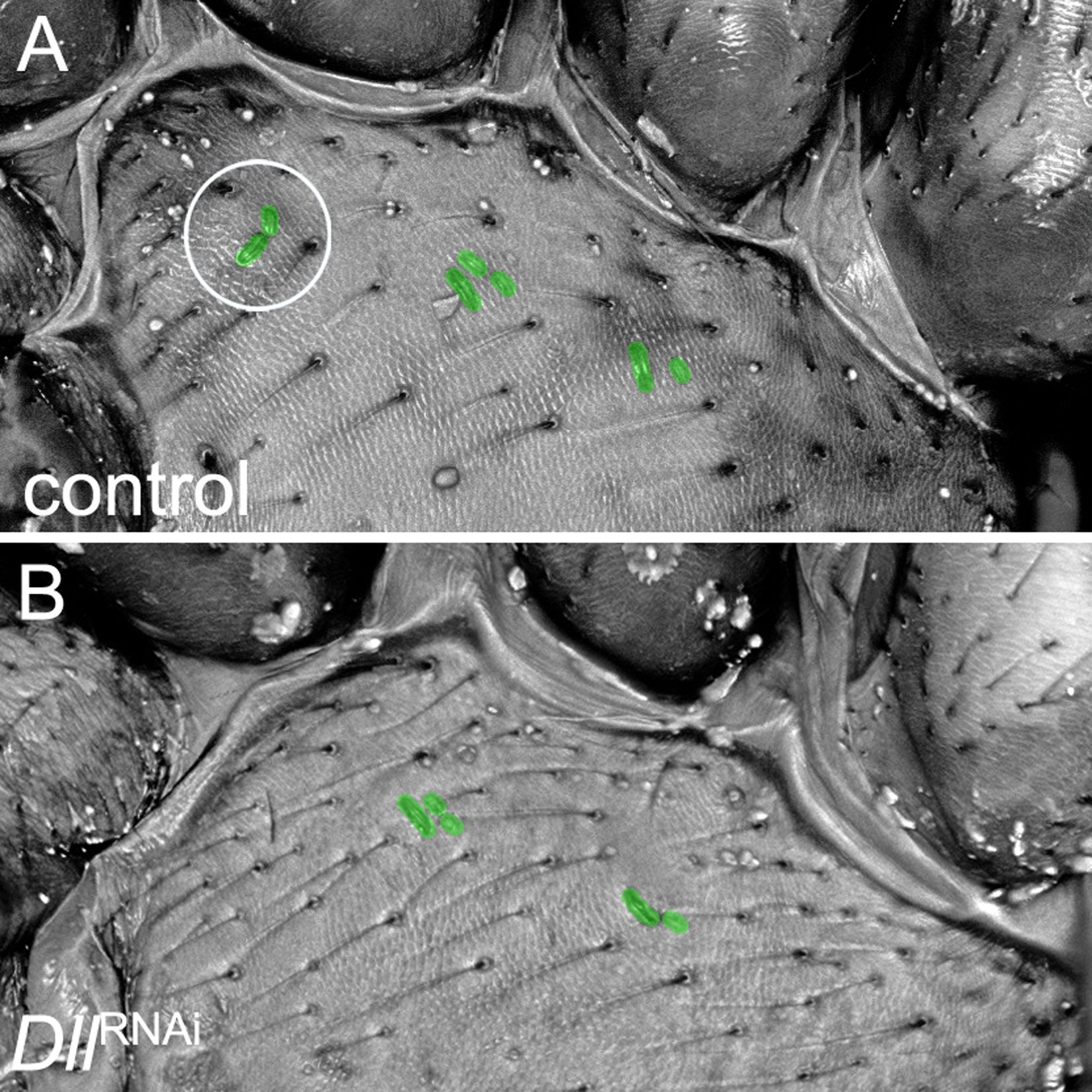

Supplement: Figure S2 — Slit sense organs on the sternum of adult male animals. (A) Control animal. (B) Dll RNAi animal. The slit sense organs are emphasized in green colour. The sense organ of the first walking leg segment (denoted by the white circle) is lacking in At-Dll RNAi animals. (TIF) [file pgen.1002342.s002.tif]

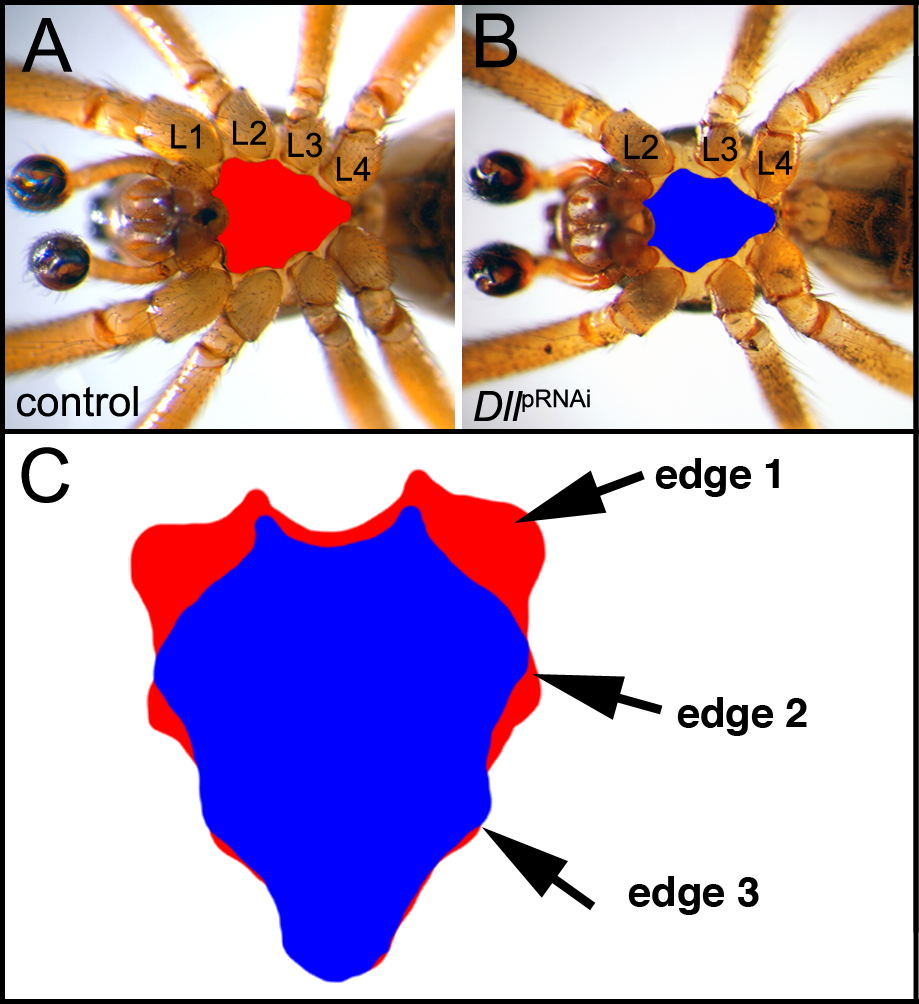

Supplement: Figure S3 — Shape comparison of the sternum in wildtype and Dll RNAi animals. (A, B) Adult wildtype and Dll RNAi spiders (same images as in Figure 2G, 2H) where the sternum has been marked in colour (red and blue, respectively). (C) Alignment of the shape of the Dll RNAi sternum (blue) with the wildtype sternum (red) reveals that the posterior edges (edges 2 and 3) align well, but edge 1 is only present in the wildtype sternum. Abbreviations: L, walking leg. (TIF) [file pgen.1002342.s003.tif]

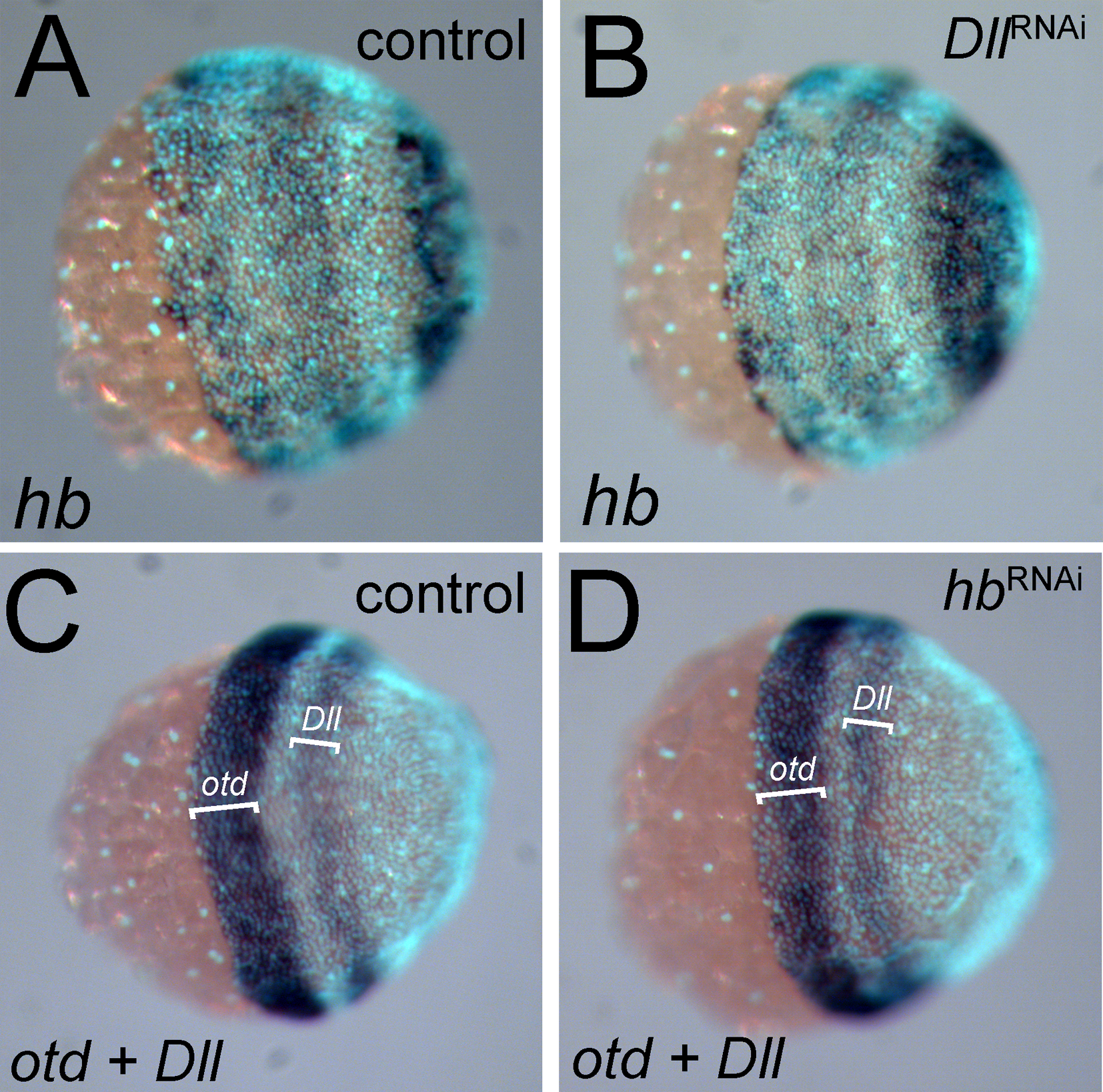

Supplement: Figure S4 — No interaction between At-Dll and At-hb. (A, B) Early (stage 6) expression of At-hb is virtually identical in control (A) and At-Dll RNAi animals (B). (C, D) Early (stage 6) expression of At-Dll is virtually identical in control (C) and At-hb RNAi animals (D). The embryos have also been stained with a probe against orthodenticle (At-otd) to mark the anterior rim of the embryo. Abbreviations: Ch, cheliceral segment; L, walking leg segment; Pp, pedipalpal segment. (TIF) [file pgen.1002342.s004.tif]

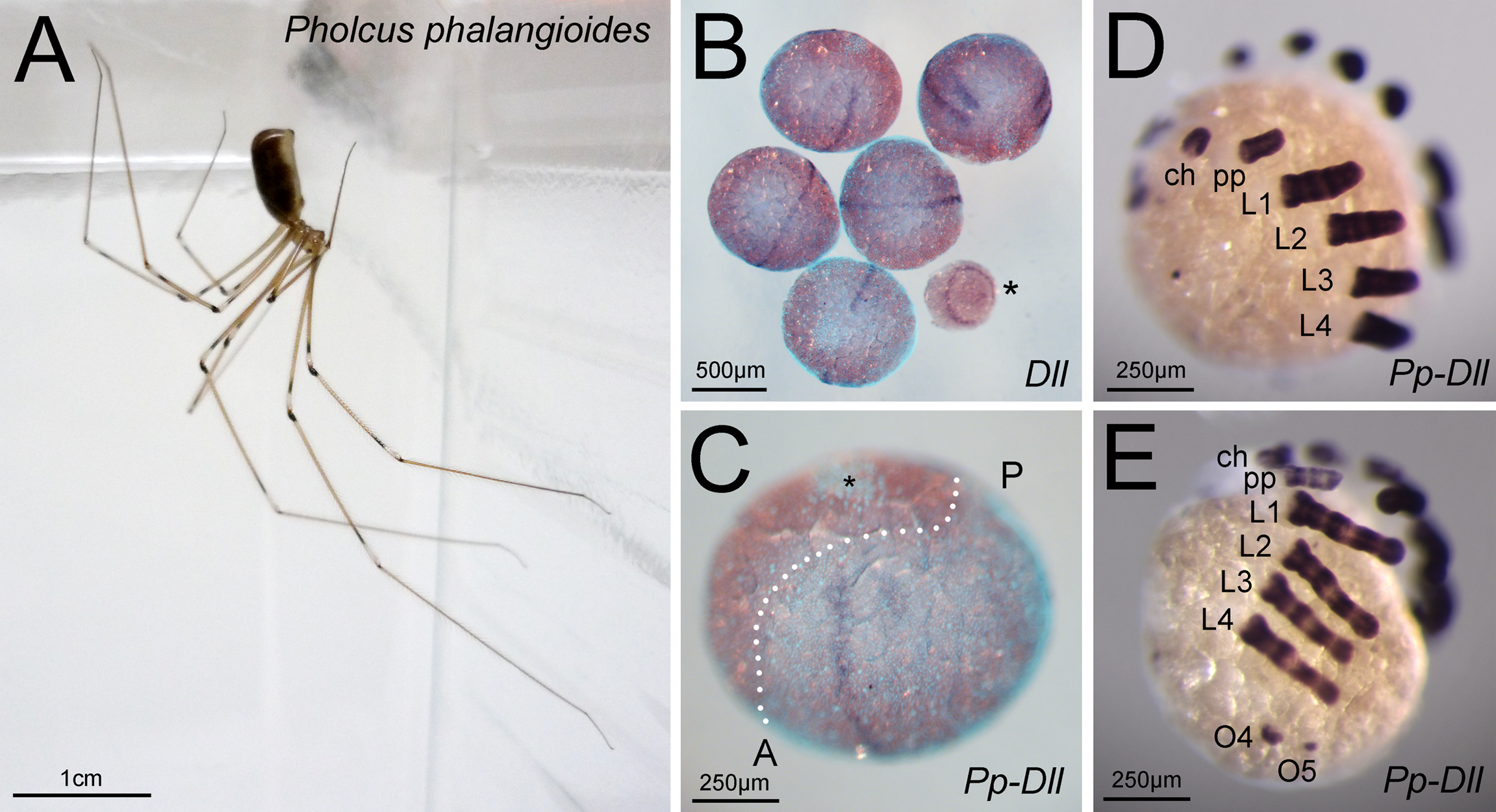

Supplement: Figure S5 — Dll expression in Pholcus phalangioides embryos. (A) An adult Pholcus phalangioides female. (B) Several embryos of P. phalangioides stained for Pp-Dll transcripts. The embryos are in a developmental stage that is comparable to stage 6/7 of Achaearanea tepidariorum embryos. For comparison, an embryo of A. tepidariorum at stage 6 (denoted by the asterisk) and stained for At-Dll transcripts has been placed next to the P. phalangioides embryos. Similar to the expression of Dll in A. tepidariorum also Pp-Dll is expressed in an early anterior ring/stripe like domain. (C) Close-up of a Pp-Dll stained P. phalangioides embryo. The asterisk marks the cumulus and the white dotted line indicates the border between the embryonic and the extra-embryonic cells. Note the Pp-Dll expression stripe that is close to the anterior end of the embryo. (D, E) Late embryonic Pp-Dll expression pattern at the beginning of germ band inversion (D) and at the end of inversion (E). Pp-Dll transcripts are detected in the distal parts of all developing appendages. This late expression is virtually identical to Dll expression of other spider species [38], [44], [50]. Abbreviations: ch, cheliceral segment; L, walking leg segment; pp, pedipalpal segment; O, opisthosomal segment; A, anterior end; P, posterior end. (TIF) [file pgen.1002342.s005.tif]

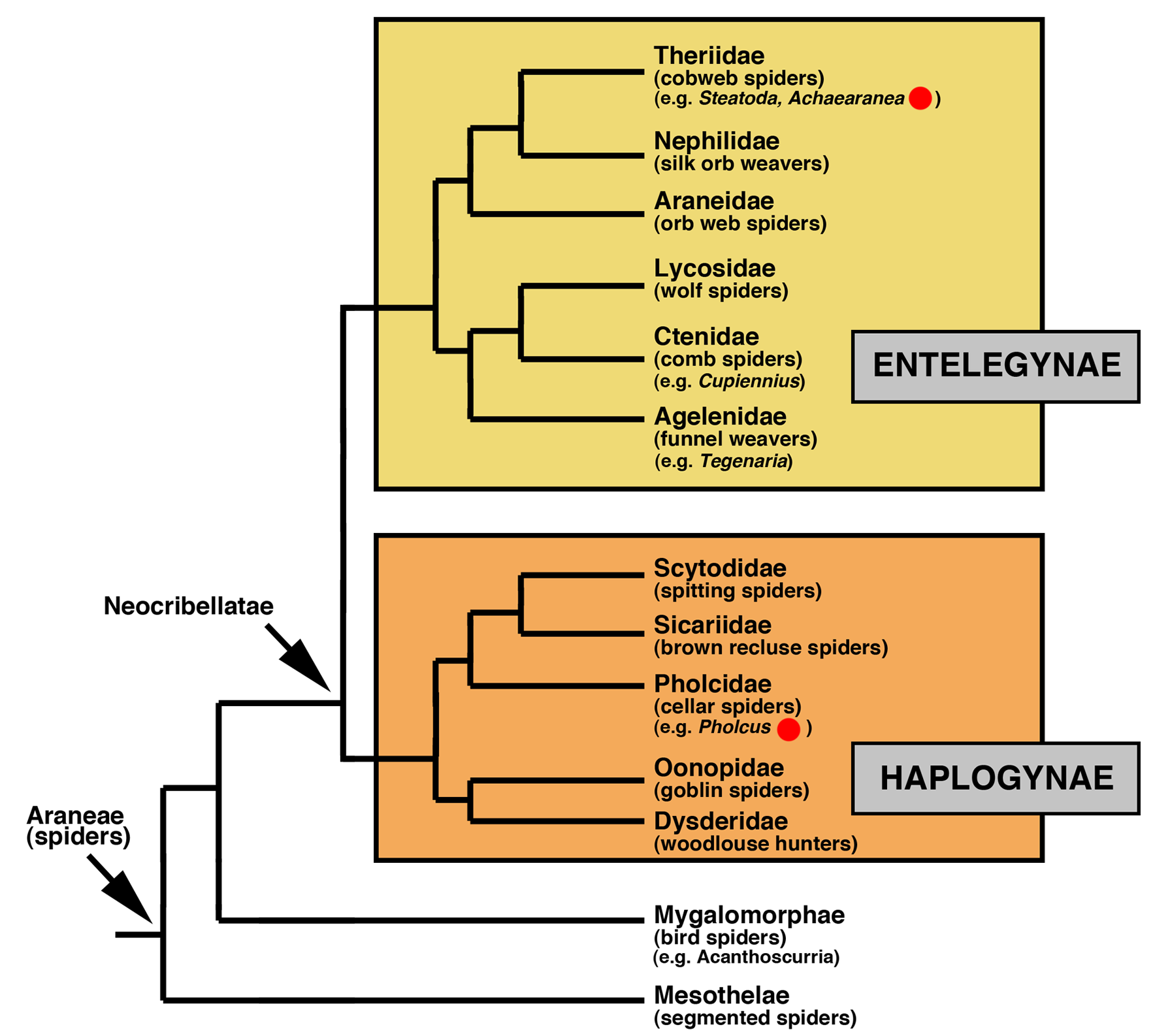

Supplement: Figure S6 — Overview of spider phylogeny. Spiders (Araneae) comprise a few primitive clades (Mesothelae, Mygalomorphae) and the speciose clade Neocribellatae that comprises the vast majority of all extant spiders. This clade consists of two sister groups, Haplogynae and Entelegynae. So far, all spider species (except for Acanthoscurria geniculata [44]) used for gene expression or gene function studies belong to the Entelegynae. The cellar spider Pholcus phalangioides is the only studied member of the Haplogynae. The similarities in early Dll expression in A. tepidariorum and P. phalangioides indicate that this patterning function of Dll was already present before the split of Haplogynae and Entelegynae. The tree is simplified after [51]. Only representative well-known families (where English common names were available) are shown, and examples (genus names) are given when representatives of the group have been used in gene expression/function studies. The red dots denote the placement of Achaearanea and Pholcus in the phylogenetic tree. (TIF) [file pgen.1002342.s006.tif]

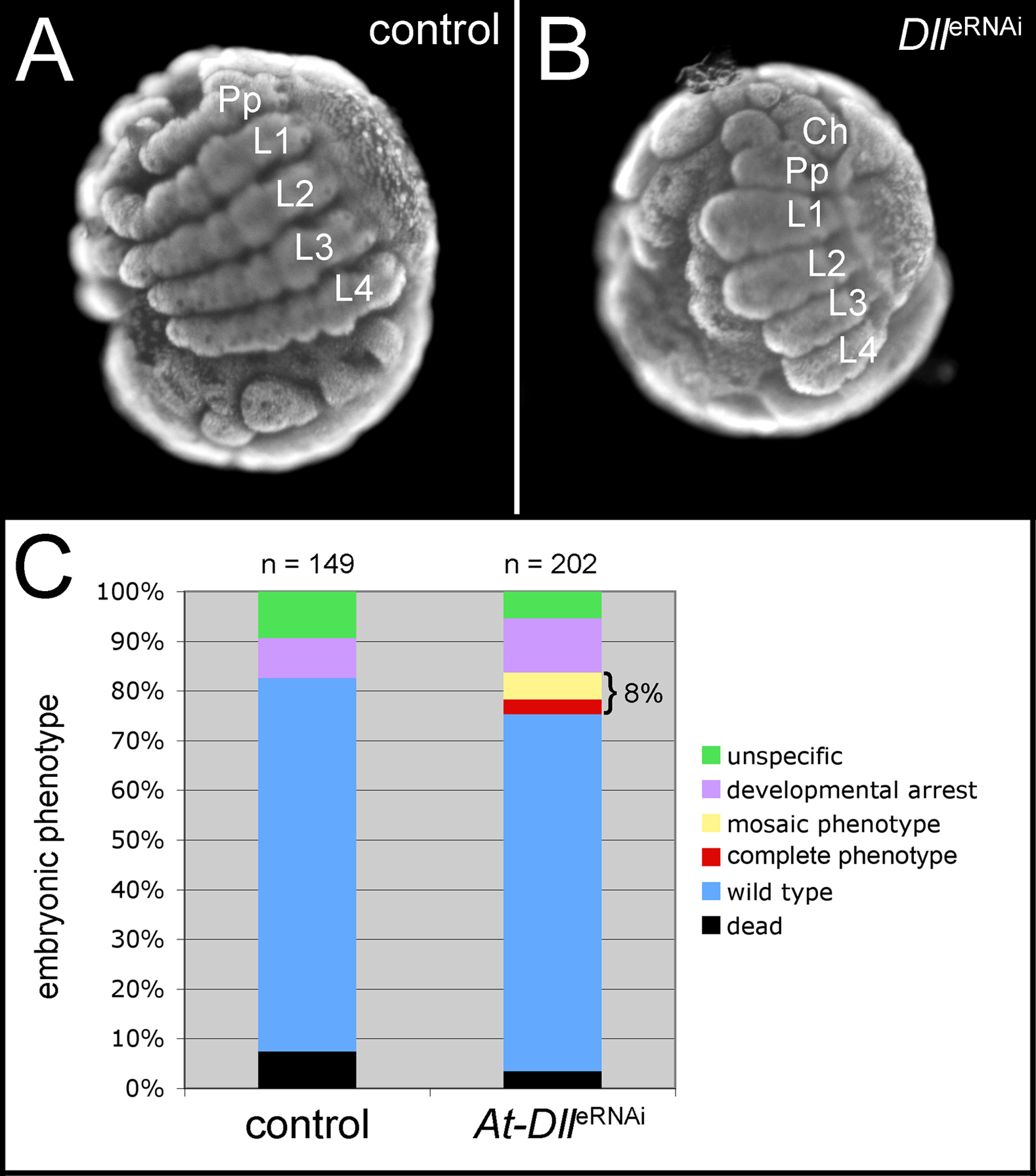

Supplement: Figure S7 — Embryonic RNA interference with At-Dll. (A) Control embryo injected with water. (B) After embryonic RNAi (eRNAi) with At-Dll all appendages are present but reduced in size. While parental RNA interference with At-Dll affects the early (segmental) and late (appendage) gene function (compare to Figure 2), embryonic RNAi only affects the development of the appendages. This result is consistent with the findings in Cupiennius salei in which eRNAi with Cs-Dll is leading to appendage defects, but no segment loss has been observed [38]. (C) Summary of the eRNAi results in A. tepidariorum. The phenotype was assessed at the late inversion stage. “Complete phenotype” refers to embryos with all appendages reduced in size. “Mosaic phenotype” refers to embryos with only some appendage reduced in size. “Developmental arrest” refers to embryos that showed some development, but died before reaching the late inversion stage, whereas “dead” refers to embryos that died at stage 4 and thus at or shortly after injection. “Unspecific” refers to embryos that reached the late inversion stage, but showed injection artefacts already known from eRNAi experiments in Cupiennius salei (i.e. malformation of body parts caused by unintentional injuries during injection). Abbreviations: Ch, cheliceral segment; L, walking leg segment; Pp, pedipalpal segment. (TIF) [file pgen.1002342.s007.tif]

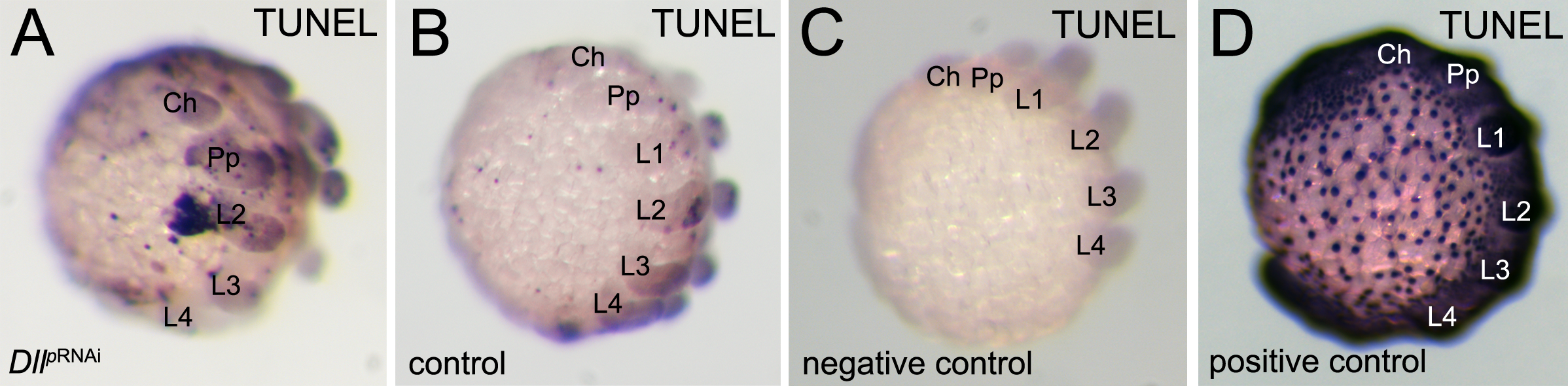

Supplement: Figure S8 — Confirmation of specific TUNEL staining after parental At-Dll knockdown. (A) An At-Dll pRNAi embryo showing enhanced cell death within the affected L1/L2 region. Please note that the L1 segment is already deleted in this embryo. (B) Only a few apoptotic cells are marked in the wild type control TUNEL staining. (C) In the negative control water instead of terminal deoxynucleotidyl transferase was added to the labelling reaction. (D) In the positive control the embryos were treated with DNase I before dig-UTP labelling [49]. Abbreviations: Ch, cheliceral segment; L, walking leg segment; Pp, pedipalpal segment. (TIF) [file pgen.1002342.s008.tif]
