# Supplementary material for: Novel Function of Distal-less as a Gap Gene during Spider Segmentation
Source: PLoS Genet. 2011 Oct 20;7(10):e1002342. doi: 10.1371/journal.pgen.1002342 (PMC3197691; doi:10.1371/journal.pgen.1002342)
Supplement: Table S1 — Summary of the At-Dll RNAi phenotypes. Adult females were injected five times (over a period of 10 days) with 1.5 µl dsRNA (3.8 µg/µl). Control females were treated the same as experimental animals, but injected with water. Female A. tepidariorum produce several cocoons during their lifetime. For this summary, embryos of cocoons 2 and 3 have been pooled and randomly chosen embryos have been assessed in detail for morphology and At-Dll expression. (DOC) [file pgen.1002342.s009.doc]

|  | wildtype | L1 missing, L2-L4 not truncated | L1 missing, L2-L4 truncated | L1 + L2 missing, L3-L4 not truncated | L1 + L2 missing, L3-L4 truncated | not developed | n |
| --- | --- | --- | --- | --- | --- | --- | --- |
| control  cocoon #2+3 | 80% | 0% | 0% | 0% | 0% | 20% | 148 |
| *At-Dll* pRNAi  cocoon #2+3 | 1% | 18% | 53% | 1% | 11% | 16% | 148 |
